# [Retracted] Inhibition of autophagy attenuated curcumol‑induced apoptosis in MG‑63 human osteosarcoma cells via Janus kinase signaling pathway

**DOI:** 10.3892/ol.2025.14907

**Published:** 2025-01-27

**Authors:** Chuan Zhang, Li-Min Wang

Oncol Lett 14: 6387–6394, 2017; DOI: 10.3892/ol.2017.7010

Following the publication of the above paper, it was drawn to the Editor's attention by a concerned reader that certain of the flow cytometric data shown in Fig. 2A on p. 6390 had already been submitted for publication in an article submitted to the same journal that was written by different authors. Moreover, there were also apparently duplicated data featured both within the cellular morphological data shown in Fig. 2C, and in certain of the western blots (both control blots and those of the proteins of interest) comparing among Figs. 2, 3 and 4, in all cases where different experimental conditions were reported.

Owing to the fact that the contentious data in the above article had already been submitted for publication prior to the submission of this article to *Oncology Letters*, and due to the apparently incorrect assembly of the data in several of the figures, the Editor has decided that this paper should be retracted from the Journal. After having been in contact with the authors, they accepted the decision to retract the paper. The Editor apologizes to the readership for any inconvenience caused.

